# A Hearing-Model-Based Active-Learning Test for the Determination of Dead Regions

**DOI:** 10.1177/2331216518788215

**Published:** 2018-07-19

**Authors:** Josef Schlittenlacher, Richard E. Turner, Brian C. J. Moore

**Affiliations:** 1 Department of Experimental Psychology, University of Cambridge, UK; 2 Department of Engineering, University of Cambridge, UK

**Keywords:** Bayesian active learning, dead region, hearing test, hearing model

## Abstract

This article describes a Bayesian active-learning procedure for estimating the edge frequency, *f*_e_, of a dead region, that is, a region in the cochlea with no or very few functioning inner hair cells or neurons. The method is based on the psychophysical tuning curve (PTC) but estimates the shape of the PTC from the parameters of a hearing model, namely *f*_e_, and degree of outer hair cell loss. It chooses the masker frequency and level for each trial to be highly informative about the model parameters in the context of previous data. The procedure was tested using 14 ears from eight subjects previously diagnosed with high-frequency dead regions. The estimates of *f*_e_ agreed well with estimates obtained using “Fast PTCs” or more extensive measurements from an earlier study. On average, 33 trials were needed for the estimate of *f*_e_ to fall and stay within 0.3 Cams of the final “true” value on the equivalent rectangular bandwidth-number scale. The time needed to obtain a reliable estimate was 5 to 8 min. This is comparable to the time required for Fast PTCs and short enough to be used when fitting a hearing aid. Compared with Fast PTCs, the new method has the advantage of using yes-no judgments rather than continuous Békésy tracking. This allows the slope of a subject’s psychometric function and thus the reliability of his or her responses to be estimated, which in turn allows the test duration to be adjusted so as to achieve a given accuracy.

## Introduction

Cochlear hearing loss is often associated with damage to the hair cells (Borg, Canlon, & Engström, 1995; Engström, 1983; Schuknecht & Woellner, 1953; see also Moore, 2007). Outer hair cell loss (OHCL) reduces the gain of the cochlear amplifier, while inner hair cell loss (IHCL) reduces the number of action potentials evoked in the auditory nerve. The total amount of cochlear hearing loss, HL_total_, can be modeled as the sum of OHCL and IHCL, when all three are expressed in dB (Moore & Glasberg, 2004). Usually, OHCL is much larger than IHCL. In some cases, there may be a region in the cochlea where the inner hair cells or neurons are functioning so poorly that a pure tone producing peak vibration in that region is detected by off-place listening (also called off-frequency listening). In other words, the tone is detected at a place whose characteristic frequency (CF) is different from the frequency of the tone. Such a region is known as a *dead region* (DR; Moore, 2001, 2004). The most common type of DR is a basal DR, which starts at a place in the cochlea with CF = *f*_e_ and extends upwards from *f*_e_. Diagnosing the presence of a basal DR, and estimating *f*_e_ is relevant to the fitting of hearing aids, including cases where a hearing aid is combined with a cochlear implant (Moore, 2001; Moore & Malicka, 2013; Zhang, Dorman, Gifford, & Moore, 2014). It may also be relevant to deciding whether a hearing-impaired (HI) person is a candidate for a cochlear implant (Moore, Glasberg, & Schlueter, 2010). This article describes a novel, behavioral method of estimating *f*_e_ that is both time-efficient and accurate.

As described earlier, a pure tone whose frequency falls in a DR may be heard because of off-frequency listening: The excitation evoked in the cochlea spreads to CFs away from the signal frequency, and if the tone is sufficiently intense, this can lead to audible excitation in an adjacent non-DR. This makes exact determination of *f*_e_ difficult. Even if there is a sharp boundary between functioning and nonfunctioning inner hair cells, the threshold for detecting a tone may increase only gradually as its frequency traverses the boundary.

A well-established test for DRs is the TEN(HL) test (Moore, Glasberg, & Stone, 2004). The task is to detect a pure-tone signal in a broadband masker, threshold-equalizing noise (TEN). The TEN has approximately a constant level per ERB_N_, where ERB_N_ is the equivalent rectangular bandwidth of the auditory filter for listeners with normal hearing (Glasberg & Moore, 1990). The masked threshold, expressed as the signal level relative to the level of the TEN in a 1-ERB_N_-wide band at 1 kHz (denoted the signal-to-noise ratio [SNR]), is about 0 dB for listeners with normal hearing, that is, the signal needs to have the same level as the TEN produces at the output of the auditory filter centered at the signal frequency. For HI listeners without DRs, the threshold for detecting the signal in the TEN is typically about 3 dB higher. However, if the frequency of the signal falls well inside a DR, it will only be detected if it evokes sufficient excitation in the non-DR below *f*_e_, and this usually requires an SNR of 10 dB or more (Moore et al., 2004; Moore, Huss, Vickers, Glasberg, & Alcántara, 2000). Hence, a DR at the signal frequency is diagnosed when the SNR at threshold is 10 dB or more (with the additional condition that the signal level at the masked threshold should be at least 10 dB above the absolute threshold). The TEN test allows rapid detection of a DR, but it does not provide a precise estimate of *f*_e_. For example, for a basal DR, if the frequency of the tone is only a little above *f*_e_, the SNR at threshold may be only a little above the “normal” value of 0 dB (Moore, 2001, 2004), and the DR will be “missed”. For a basal DR, the “true” value of *f*_e_ usually lies somewhat below the lowest signal frequency at which the TEN-test criteria are met.

Another test for DRs is based on psychophysical tuning curves (PTCs; Chistovich, 1957). Here, the task is to detect a pure-tone signal in the presence of a narrowband noise. The frequency ( *f*_sig_) and level (*L*_sig_) of the signal are fixed, and the center frequency and level of noise ( *f*_mask_, *L*_mask_) are varied. A PTC is the value of *L*_mask_ required to mask the signal plotted as a function of *f*_mask_. For normally hearing subjects and subjects with hearing loss but without DR, the PTC has a minimum near *f*_sig_, that is, where the frequencies of the signal and noise coincide. When the signal frequency falls within a DR, the noise only needs to mask the part of the excitation pattern that falls outside the DR. Thus, the minimum in the PTC occurs when *f*_mask_ ≈ *f*_e_. PTCs measured using several discrete values of *f*_mask_ have been used to determine *f*_e_ in experimental studies (Kluk & Moore, 2005; Moore & Alcántara, 2001; Moore et al., 2000). However, this method is too time-consuming for use in clinical practice.

A method of obtaining PTCs more rapidly is called “Fast PTCs” (Sęk, Alcántara, Moore, Kluk, & Wicher, 2005). The method uses Békésy tracking: *f*_sig_ and *L*_sig_ are kept constant, and the signal is continuously pulsed on and off, while *f*_mask_ is continuously increased (or decreased) over time. The subject presses a button to indicate whether he or she hears the signal, and *L*_mask_ is increased while the button is pressed and decreased while it is released. This yields a PTC within 3 to 6 min, depending on the selected range of *f*_mask_ and the rate of change of *f*_mask_. However, the method has the disadvantage that the subject may “lose track” of the signal; essentially, the subject forgets what to listen for. For some subjects, many Fast PTCs using several values of *f*_sig_ and *L*_sig_ are needed to obtain a robust estimate of *f*_e_ (Kluk & Moore, 2005, 2006).

The present article describes a PTC-based method that is fast but also accurate. The masked threshold of a pure tone in noise, and thus a PTC, is predicted using a hearing model (Moore & Glasberg, 2004) based on the simple assumption that the signal can be heard if its excitation exceeds the excitation evoked by the noise at any CF and cannot be heard if its excitation is below the excitation evoked by the noise or below the audiometric threshold at all center frequencies. The calculated excitation patterns of the tone and noise depend on their frequency and level and the subject’s hearing loss. The model parameters used to characterize the hearing loss are HL_total_, OHCL, and *f*_e_. HL_total_ is known from the audiogram. In practice, when *f*_sig_ falls above *f*_e_, the maximum excitation evoked by the signal always occurs at *f*_e_ and the highest ratio of signal excitation to masker excitation occurs at *f*_e_. Therefore, the value of OHCL only needs to be estimated at *f*_e_; this value is denoted OHCL( *f*_e_). Responses obtained using various combinations of *f*_mask_ and *L*_mask_ limit the possible combinations of *f*_e_ and OHCL( *f*_e_).

In the proposed method, a probabilistic approach is used to express the degree of “belief” about the values of *f*_e_ and OHCL( *f*_e_). The belief is represented by a probability distribution over *f*_e_ and OHCL( *f*_e_), *p*( *f*_e_, OHCL( *f*_e_)), that indicates how likely each setting of *f*_e_ and OHCL( *f*_e_) is, given the data. As the test proceeds, *p*( *f_e_*, OHCL( *f_e_*)) collapses to a single point, that of the true parameters. To make the test fast, *f*_mask_ and *L*_mask_ are chosen to be highly informative about *f*_e_ and OHCL( *f*_e_). A similar approach was used by Kontsevich and Tyler (1999) to estimate a psychometric function, its mean and slope being the model parameters. For each trial, the stimulus level was chosen to minimize the expected entropy (Shannon, 1948) of the model parameters given the previous data (see the Method section for details). The psychometric function has only a single stimulus parameter, the level. Other work in cognitive science has made this strategy tractable when two or more parameters need to be estimated (DiMattina, 2015; Houlsby, Huszár, Ghahramani, & Lengyel, 2011; Houlsby et al., 2013; Kujala & Lukka, 2006), by using equivalent but computationally less expensive formulae. Active-learning paradigms that maximize the information gained on each trial have been used in the auditory domain to estimate the bandwidth of the auditory filter (Shen & Richards, 2013) and to estimate the audiogram (Song, Sukesan, & Barbour, 2018; Song et al., 2015). In this article, we show that the proposed Bayesian active-learning procedure determines *f*_e_ accurately within minutes. It is thus short enough to be applied in clinical practice.

## Method

### Overview

First, two audiograms were obtained for each test ear of each subject. One was obtained using an audiometer and the manual procedure recommended by the British Society of Audiology (2011). The other was obtained using an active-learning procedure similar to that described by Song et al. (2015). On average, the threshold obtained using the active-learning method was 3.2 dB below that obtained using the audiometer (standard deviation of the difference: 6.7 dB). The variability is comparable to that reported by Song et al. (2015). The audiograms obtained using the active-learning procedure were used for further analysis since they were obtained on the same apparatus and yielded a continuous estimate of the threshold as a function of frequency.

It has been shown that the likelihood of a DR being present at a given frequency increases markedly when HL_total_ at that frequency exceeds 65 dB HL (Vinay & Moore, 2007). Hence, in a second step, the frequencies at which the audiometric threshold reached 65, 70, and 75 dB HL ( *f*_65_, *f*_70_, and *f*_75_) were extracted, and a quick version of the TEN(HL) test was used to assess whether *f*_65_ fell in a DR (see later for details). If it did not, the quick TEN(HL) test was repeated to assess whether *f*_70_ and *f*_75_ fell in a DR. Finally, each subject was tested using a Bayesian active-learning procedure (called “Smart DRT”) to estimate *f*_e_, and a Fast PTC was also obtained. Since the frequency at the tip of the Fast PTC is usually taken as an estimate of *f*_e_, one can think of the goal of the Smart DRT as being to estimate the frequency at the tip of the PTC but without determining the entire PTC. To assess the repeatability of the Smart DRT, it was administered three times for each test ear. Half of the ears were tested using the Smart DRT before the Fast PTC, and the other half were tested in the reverse order.

For all tests, the subject was asked to indicate whether the signal was present on each trial or not, that is, a yes-no task was used. This was chosen in preference to a two-interval forced-choice task since random guessing in a two-interval forced-choice means that on average, a correct response will be given on half of trials where the signal was not heard at all, whereas a “yes” response in a yes-no task probably indicates that the subject did obtain some sensory evidence indicating the presence of the signal and is therefore more informative (Green & Sweets, 1974). In most cases, subjects adopted a cautious response criterion, usually responding “no” on trials where the signal was absent.

### Subjects

**Figure 1.**
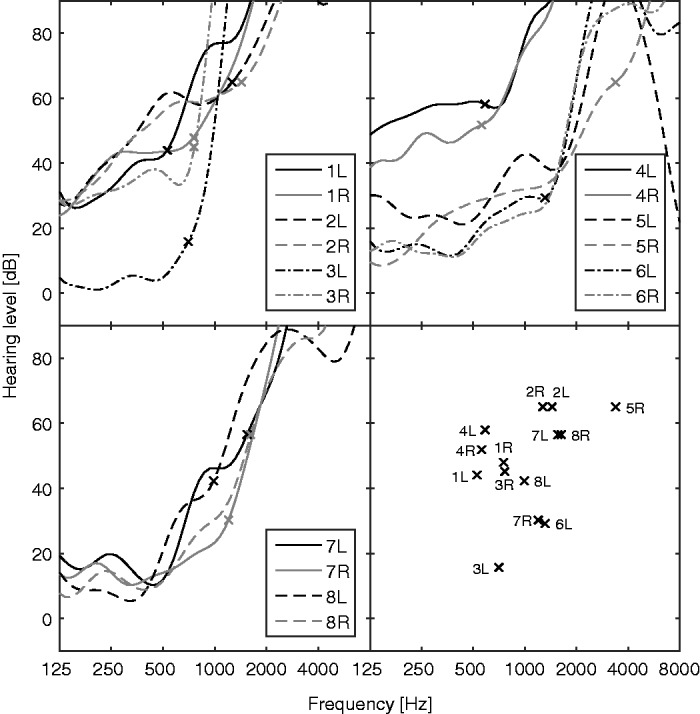
Audiograms obtained using the active-learning method for the 14 test ears and the two contralateral ears without DR of the same subjects (5L and 6R). Lines show HL_total_, and crosses show the values of *f*_e_ that were determined by the Smart DRT. The two upper panels and the lower left panel show results for subsets of ears, and the bottom right panel shows the values of *f*_e_ plotted against the audiometric threshold at *f*_e_ for all ears with DRs.

Fourteen ears from seven male subjects and one female subject aged 45 to 82 years (mean 72 years) were tested. All test ears had previously been diagnosed as having extensive basal DRs using the TEN(HL) test and Fast PTCs, as described by Salorio-Corbetto, Baer, and Moore (2017). Six of the subjects had DRs in both ears, and two had a DR in one ear only. The contralateral ears of these two subjects had impaired hearing but no DR. All subjects had normal hearing to moderate hearing loss at low frequencies and hearing loss of more than 65 dB at high frequencies. The slope of the hearing loss varied across subjects. At a hearing loss of 65 dB HL, the derivative of HL_total_ ranged from 14 dB/octave to 145 dB/octave. The audiograms for all test ears, including the two without a DR, are shown in Figure 1, with lines denoting HL_total_ and crosses the values of *f*_e_ that were determined by the Smart DRT. In addition, the quick TEN(HL) test was conducted using two normal-hearing and two HI subjects without a DR, in order to confirm that the quick TEN(HL) test did not falsely identify DRs when none were present.

### Apparatus

For all tests except standard audiometry, stimuli were generated digitally at a sample rate of 48 kHz using a personal computer. The signal was converted to analog form by an M-Audio Delta 44 audio interface (Cumberland, RI) and presented via a Sennheiser HDA200 headset (Wedemark, Germany). The frequency response of the headset at the eardrum was estimated using KEMAR (Burkhard & Sachs, 1975) and used to determine sound levels at the eardrum. The traditional audiogram was measured using a Grason-Stadler GSI-61 audiometer and Telephonics TDH-50 headphones.

### Stimuli

All signals were presented monaurally. For the quick TEN(HL) test, a TEN(HL) noise was generated in the same way as described by Moore et al. (2004), but it was designed for use with Sennheiser HDA200 headphones. It was band limited between 354 and 6500 Hz, and its level is specified in dB HL/ERB_N_ at 1 kHz. The noise had a low crest factor, to allow presentation at high levels without peak clipping. However, after passing through the auditory filters, the noise would have had a Gaussian distribution of instantaneous amplitudes.

The task in the Smart DRT was similar to that for a traditional PTC, that is, to detect a pure tone in the presence of bandpass-filtered noise. The bandwidth of the masking noise was 1 ERB_N_ or 200 Hz, whichever was greater. The bandwidth was selected to minimize the influence of beats on the masked threshold (Kluk & Moore, 2004, 2005). The level (*L*_mask_) and center frequency ( *f*_mask_) of the noise were varied. The noise had a flat spectrum within the passband and was generated using an inverse discrete Fourier transform, the spectrum being defined with a resolution of 1 Hz. A broadband TEN with a level of 35 dB HL/ERB_N_ was added to mask potential distortion products (Alcántara & Moore, 2002).

The temporal envelopes of the stimuli were the same for the quick TEN(HL) test and the Smart DRT. The signal consisted of three pulses of a tone with Hann-windowed rise and fall times of 20 ms and a total duration of 150 ms each. The interval between pulses was 100 ms. The noise was switched on 100 ms before the first pulse started and switched off 100 ms after the last pulse finished, again with Hann-windowed rise and fall times of 20 ms, leading to a total duration of 850 ms. The stimuli for determining Fast PTCs were generated using the software described by Sęk and Moore (2011).

### Procedure for the Quick TEN(HL) Test

The quick TEN(HL) test was conducted to determine whether the test ear had a DR at *f*_65_, *f*_70_, or *f*_75_. The value of *f*_sig_ was initially set equal to *f*_65_. *L*_sig_ was set to 75 dB HL, which is 10 dB above the absolute threshold determined using the active-learning procedure, that is, 10 dB SL. The TEN level was 65 dB HL/ERB_N_. This slightly lower TEN level than that proposed by Moore et al. (2004) was used here to avoid uncomfortably loud levels, especially for subjects with good low-frequency hearing. Initially, the signal was presented without any noise, and the subject was asked to confirm that it was audible. Thereafter, 10 trials containing the signal in TEN and 10 trials with the TEN only were presented in random order. The task was to indicate whether the signal was heard or not. Denote the proportion of trials where the signal was present and the subject responded “yes” (hits) as X and the proportion of trials where the signal was absent and the subject responded “no” (correct rejections) as Y. The proportion of false alarms (responses of “yes” when the signal was absent) is 1 − Y. If the signal is not detectable at all, X = 1 − Y. We chose as a threshold criterion X ≥ 1.5(1 − Y), that is, X + Y ≥ 1.5. Given that there were 10 trials of each type, this corresponds to a total number correct ≥15. In practice, the scores were mostly close to 10 correct or (in the case of the subjects without DRs at *f*_65_) close to 20 correct. Thus, the outcome was not affected by the exact threshold criterion used.

If the subject scored 14 or fewer correct, it was assumed that there was a DR at *f*_65_ (see Moore et al., 2004). If the subject scored 15 or more correct, the quick TEN(HL) test was repeated with *f*_sig_ set to *f*_70_, *L*_sig_ set to 80 dB HL, and the TEN(HL) level set to 70 dB HL/ERB_N_. If the subject scored fewer than 15, it was assumed that there was a DR at *f*_70_. If the subject scored 15 or more, the quick TEN(HL) test was repeated with *f*_sig_ set to *f*_75_, *L*_sig_ set to 85 dB HL, and the TEN(HL) level set to 75 dB HL/ERB_N_. The signal was never detected in this case, indicating the presence of a DR at *f*_75_. The value of *f*_sig_ used for the Smart DRT and Fast PTCs was taken as the lowest frequency (out of *f*_65_, *f*_70_, and *f*_75_) at which the subject scored less than 15 correct. The signal level was always 10 dB SL.

### Procedure for the Smart DRT

A single trial in the Smart DRT included three intervals. The task was to indicate whether a pure-tone signal was present in the third interval or not. For four practice trials and 100 subsequent trials, the intervals contained the signal alone, the noise alone, and the signal plus the noise, in that order. For 20 trials that were randomly mixed with the other 100 trials, the signal was omitted from the third interval, to assess false positives.

The procedure started with four easy practice trials to introduce the subject to maskers with different frequencies and levels. The experimenter inspected the results and could intervene if the subject performed unexpectedly badly. These four trials were not considered for any further analysis. The next 16 trials were chosen by simple rules: Initially, *f*_mask_ was set 2 Cams below *f*_sig_, where Cams are units of the ERB_N_-number scale (Moore, 2012). *L*_mask_ was increased across successive trials, with values of −20, −9, −6, −3, 0, 3, 6, and 9 dB relative to *L*_sig_. This was then repeated with *f*_mask_ set 4 Cams below *f*_sig_. This initial grid was intended to familiarize the subject with trials of varying detectability. Subsequent values of *L*_mask_ and *f*_mask_ were chosen by a model-based active-learning method. The responses for the initial 16 trials were taken into account by the active-learning method. Doing so limited the impact of a wrong response in the early active-learning trials.

There were two free parameters in the model. The value of *f*_e_ was one of them. It was assumed that when *f*_sig_ fell within a DR, detection of the signal would depend on the relative response to the masker and signal at the output of the auditory filter tuned to *f*_e_, that is, on the relative excitation levels evoked by the signal and masker at *f*_e_. The output of this filter was calculated using the method described by Moore and Glasberg (2004). The sharpness of the filter was assumed to be determined by the total hearing loss at *f*_e_, denoted HL_total_( *f*_e_), the amount of hearing loss at *f*_e_ that was attributed to reduced outer hair cell function, OHCL( *f*_e_), and the spectrum of the input. The value of HL_total_( *f*_e_) was based on the audiogram obtained using the active-learning procedure. The value of OHCL( *f*_e_) was a second free parameter.

For given values of *f*_e_ and OHCL( *f*_e_), the excitation levels evoked by the noise (*E*_noise_) and by the signal (*E*_sig_) were calculated. *E*_noise_ was substituted by the maximum of *E*_noise_ and HL_total_( *f*_e_), because the threshold in quiet could limit the audibility of the signal. The difference between *E*_sig_ and *E*_noise_ was taken as an indicator of the audibility of the signal. To provide a very simple model for the audibility of the signal in noise, it was assumed that when the difference was 0 dB (*E*_sig_ = *E*_noise_), the probability of a correct yes response (a hit, denoted *y* = 1) was 50%. The probability of a hit was calculated from a Gaussian cumulative density function (CDF) with a standard deviation of 3 dB

(1) $$p(y=1|x,\theta )=\mathrm{CDF}(E_{\text{sig}}(\theta )-E_{\text{noise}}(\theta ,x),\mu =0,\sigma =3)$$

where *x* denotes the presentation parameters *f*_mask_ and *L*_mask_, and θ denotes the model parameters *f*_e_ and OHCL( *f*_e_). A standard deviation of 3 dB was thought to be a reasonable estimate of the slope of the psychometric function for detection of a tone in noise (Green, Birdsall, & Tanner, 1957). In general, if the standard deviation is assumed to be smaller than it actually is, the algorithm becomes “overconfident”, that is, assigns probabilities that are too high around the currently most probable model parameters. Without additional measures, this may result in the algorithm getting “stuck” at an incorrect estimate. If, on the other hand, the standard deviation is assumed to be larger than it actually is, the algorithm becomes more “cautious” and may waste time exploring extreme parameter values. The CDF was scaled to have values between 0.01 and 0.99 to incorporate a lapse rate for accidentally pressing the “wrong” button. Doing this was thought to allow the method to recover from “wrong” responses more quickly.

The probability of obtaining the observed sequence of yes and no responses after *N* trials, given a set of model parameters and the presented stimuli, is as follows:

(2) $$p(D|x,\theta )=\overset{N}{\underset{i=1}{\Pi }}p(y_{i}|x_{i},\theta )$$

where *D* denotes the collected response data, and *i* denotes the *i*th trial. However, we are not interested in the probability of the collected data given some assumed model parameters, but in the probability of the model parameters, in particular *f*_e_, given the collected data. The probability of a set of model parameters given the collected data can be obtained by Bayes’ rule and is as follows:

(3) p(θ|D,x)∝p(D|θ,x)p(θ)

where *p*(θ) is the prior distribution of the model parameters, which does not depend on *x*. A uniform distribution was assumed for *p*(θ) with the constraint that OHCL( *f*_e_) could not be greater than HL_total_( *f*_e_). Also, the sets ( *f*_e_, OHCL( *f*_e_)) were restricted to avoid the entire audible region of the excitation pattern evoked by the signal falling above *f*_e_. If this were the case, the signal would not even be audible in quiet according to the model, but it had been established that the signal in quiet with a level of 10 dB SL was clearly audible.

The parameters x* of the next trial were chosen with a probability proportional to the mutual information *I* between the response on the next trial y* and the model parameters θ,

(4) $$I(y^{*};\theta |x^{*})=H(y^{*}|x^{*},D)-{\text{E}}_{\theta ∼p(\theta |D)}(H[y^{*}|x^{*},\theta ])$$

where *H* is the Shannon entropy (Shannon, 1948), a measure of the uncertainty in a variable. The first term is the entropy of the expected response, and the second term is the expected conditional entropy of the response given the belief about the model parameters (for more details, see Houlsby et al., 2011). Mutual information is a mathematically principled measure of the mutual dependence between variables that underpins the field of information theory, and it is used here to pick stimulus parameters. Stimuli are informative about the parameters *f*_e_ and OHCL( *f*_e_) if the outcome is uncertain, that is, when the probability of responding “yes” is close to 50% rather than close to 0 or 100% (first term of Equation 4). In our case, this term is large when the masker level is close to the threshold value. The second term is low in regions of x* where the responses have been inconsistent or where no data were collected. The net result is to explore stimuli that are close to the current threshold estimate, but distant from areas where “this” threshold estimate is confident.

Mutual information is an optimal criterion, but it can only be tractably optimized in a “greedy” manner, that is, the algorithm looks only one trial ahead and can only pick the next stimulus without considering potentially informative sets of several stimuli. Greedily selecting the stimulus that is most informative about θ can, in some circumstances, lead to failure to explore relevant regions of parameter space. Here, the failure would have been a result of the algorithm being unable to take into account the information gained on subsequent trials. This problem was avoided by picking the next stimulus with a probability proportional to the mutual information. This strategy leads to more exploration of the parameter space but still picks stimuli that are highly informative about θ.

In addition, the algorithm was “encouraged” to choose *f*_mask_ close to the current estimate of the most likely value of *f*_e_, denoted f^e, by applying an additional weight to *f*_mask_, based on a normal distribution around f^e with a standard deviation of 1.5 Cams. This was done for several reasons. First, it avoided values of *f*_mask_ for which the model predictions were likely to be less accurate. Second, it utilized the fact that the PTC shape is most distinctive around *f*_e_. Third, it favored low and thus more comfortable masker levels. Thus, the parameters for the next trial were chosen randomly with probability.

(5) p(x*=fmask,Lmask)∝I(y*;θ|x*)N(x*;f^e,σ2)

where σ = 1.5 Cams and *N*(…) denotes the normal distribution. In addition, values of *f*_mask_ within 2 Cams of the value of *f*_mask_ on the previous trial were excluded, to avoid “duplication” of information.

The belief about *f*_e_ and OHCL( *f*_e_) was updated after each trial (Equation 3), and the f^e value that was most likely after the last trial was chosen as the final estimate of *f*_e_. The computations were made tractable, so that they could be performed in the intertrial interval of about 2 s, by using numerical integration and precomputing excitation patterns before the experiment started.

### Fast PTCs

Fast PTCs were obtained for comparison with the Smart DRT results, using the software of Sęk and Moore (2011). The values of *f*_sig_ and *L*_sig_ were those determined in the quick TEN(HL) test. The masker center frequencies covered the range from one octave below *f*_sig_ to slightly above *f*_sig_. A low-pass noise with a cutoff frequency of 200 Hz and level of 40 dB SPL was added to mask possible difference tones (Alcántara & Moore, 2002). Two PTCs were obtained, one using an upward frequency sweep and one using a downward sweep with the masker level changing at a rate of 2 dB/s. The duration of each frequency sweep was 4 min. If a subject reported difficulties in the first run, for example forgetting what to listen for, or if the results were erratic, for example because the PTC did not have a convex shape or clearly showed a lapse of attention, the run was repeated. In this case, only the results from the repeated run were analyzed.

## Results

### Quick TEN(HL) Test

The two normal-hearing subjects and two HI subjects without a DR were tested on the quick TEN(HL) test using *f*_sig_ = 1 kHz, *L*_sig_ = 75 dB HL, and TEN(HL) level = 65 dB HL/ERB_N_. All scored at least 19 out of 20 trials correct. For 13 of the test ears with DRs, the signal with frequency *f*_65_ was never heard when presented at 75 dB HL in the presence of the TEN(HL) at 65 dB HL/ERB_N_, suggesting the presence of a DR at *f*_65_. For the remaining test ear (3R), the subject achieved 20 and 19 trials correct for the frequencies *f*_65_ and *f*_70_, but could not hear the signal with frequency *f*_75_ when presented at 85 dB HL in the presence of the TEN(HL) at 75 dB HL/ERB_N_, suggesting the presence of a DR at *f*_75_.

### Smart DRT

**Figure 2.**
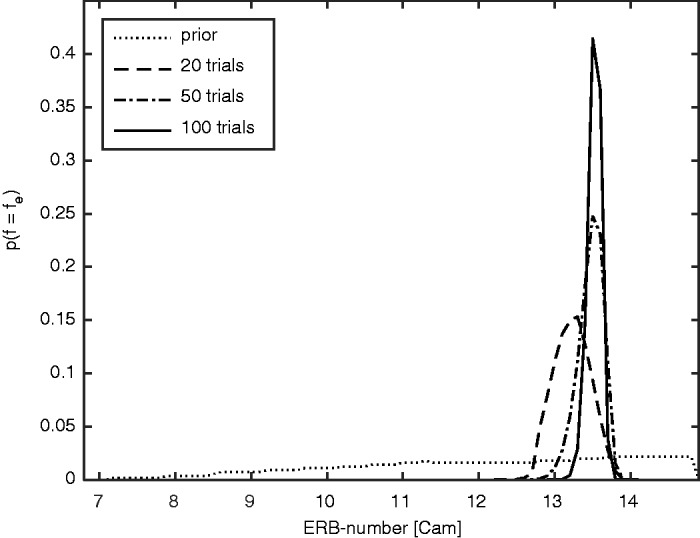
Probability density functions of the estimate of *f*_e_, using a bin width of 0.1 Cam, for the second run of ear 3R. Dotted, dashed, dash-dotted, and solid lines show the probability density functions after 0, 20, 50, and 100 trials, respectively. Catch trials are not included in the number of trials.

To illustrate the operation of the Smart DRT, Figure 2 shows the belief about *f*_e_ after 0, 20, 50, and 100 trials for the second run for ear 3 R. The probability of *f*_e_ was obtained by marginalizing the joint distribution over OHCL( *f*_e_), that is, integrating the joint probabilities over all OHCL( *f*_e_) for each *f*_e_. This averages away the uncertainty in OHCL( *f*_e_). The prior probability (dotted line), which was estimated before considering any results for the initial grid, but just taking the audiogram and model constraints into account, was approximately uniform between about 11 Cams (520 Hz) and 14.8 Cams (900 Hz, corresponding to* f*_sig_), and decreased to 0 at 7.2 Cams (270 Hz) and above 14.8 Cams (since it was already known that *f*_e_ was below *f*_sig_). The distribution narrowed with increasing number of trials, *N*, with maxima at 13.3 Cams (730 Hz) after 20 trials and at 13.5 Cams (750 Hz) after 50 and 100 trials. On average, 33 trials were needed for the estimate of *f*_e_ to fall and stay within 0.3 Cams of the final estimate, indicating that less than 20 active-learning trials after the initial grid were sufficient to produce a reliable result (see also Figure 6).

**Figure 3.**
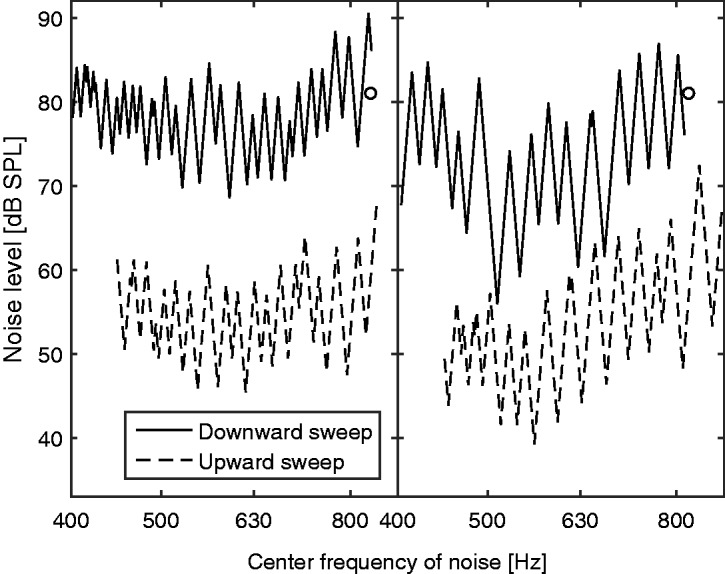
Fast PTCs for ears 4L (left) and 5R (right). The solid lines show PTCs for downward sweeps, and the dashed lines show PTCs for upward sweeps; the latter were shifted down by 25 dB for clarity. Circles show the frequency and level of the signal.

The final estimates of *f*_e_ (those obtained at the end of each run) varied across runs of the Smart DRT and across ears from 0.5 kHz (ear 1 L) to 3.3 kHz (ear 5R). To assess the accuracy of the estimates, initially the values of *f*_e_ derived from the Fast PTCs for each ear were used as a “gold standard” or “reference”. For the Fast PTCs, the value of *f*_e_ is usually taken as the frequency at the tip of the PTC, *f*_tip_ (Moore & Alcántara, 2001; Moore et al., 2000). However, the exact value of *f*_tip_ is sometimes difficult to estimate because of the jagged nature and somewhat irregular shapes of the Fast PTCs. Sęk and Moore (2011) proposed several methods for estimating *f*_tip_, which can lead to somewhat different values. Here, the reference values of *f*_e_ were calculated by interpolating the 4-point moving averages of the Fast PTCs with splines, averaging the fitted levels at each masker center frequency for the upward sweep and the downward sweep, and taking the minimum of the curve obtained in this way. The Fast PTCs for 7L, 8L, and 8R did not show clear minima, and for these ears, the mean of the estimates of *f*_e_ for the three Smart DRT runs was taken as the reference. The Fast PTCs for 7L were very flat, with the downward sweep even being slightly concave. The Fast PTCs for 8L and 8R showed one or two reversals at the start for both the downward and upward sweeps but reached the maximum masker level immediately afterwards. Example Fast PTCs for 4L and 5R are shown in Figure 3. Both PTCs were rather flat, which is typical for ears with high values of OHCL. The irregular nature of the PTCs limits the accuracy with which *f*_tip_ can be determined.

**Figure 4.**
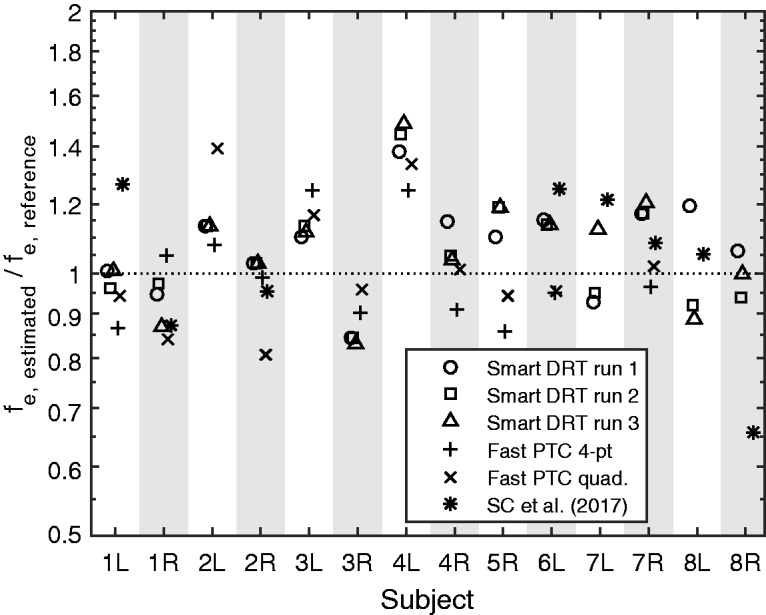
Estimated values of* f*_e_ expressed relative to a reference value, which was derived in most cases from the Fast PTCs (see text for details). Circles, squares, and triangles represent results for the three Smart DRT runs. Crosses and diagonal crosses represent two alternative measures of the tip frequency of the Fast PTCs, and asterisks show estimates obtained by Salorio-Corbetto et al. (2017) for some of the same ears as tested here.

For each test ear, the values of *f*_e_ obtained using each run of the Smart DRT were divided by the reference value of *f*_e_ for that ear. The resultant relative values of *f*_e_ are shown in Figure 4. Results for the three runs of the Smart DRT are indicated by circles, squares, and triangles. Ideally, these values should lie close to 1. In practice, they fell in the range 0.83 to 1.49 with a geometric mean of 1.06. However, the three Smart DRT estimates for each ear generally fell close to one another (except perhaps for 7L and 8L), indicating good internal consistency of the Smart DRT procedure. The range of the three Smart DRT estimates, expressed as the maximum estimate divided by the minimum estimate, had a geometric mean across ears of 1.08, and varied from 1.00 for 2L and 2R to 1.35 for 8L.

Two further estimates of *f*_tip_ were derived from the Fast PTC data (neither of which could be used with 7L, 8L, and 8R). For one, a 4-point moving average was obtained separately for the upward sweep and the downward sweep, the frequency at the minimum of each curve was found, and the two frequencies were averaged. The resulting estimates, expressed relative to the reference values of* f*_e_*,* are shown as crosses in Figure 4. For the other additional estimate, a quadratic function was fitted separately to the data for the upward sweep and the downward sweep, the frequency at the minimum of each function was found, and the two frequencies were averaged. The resulting relative estimates are shown as **×** symbols in Figure 4. If the estimates of *f*_tip_ were consistent across methods, the crosses and **×** symbols would all fall close to 1. In fact, they fell between about 0.8 and 1.4. The variability across the three estimates of *f*_tip_ derived from the Fast PTC data was generally larger than the variability across the three estimates of *f*_e_ obtained from the three runs of the Smart DRT, suggesting that at least part of the deviation of the relative values from 1 for the latter arose from errors in the reference values. The range of the three Fast PTC estimates, expressed as the maximum estimate divided by the minimum estimate, had a geometric mean across ears of 1.19, and varied from 1.05 for 6L to 1.39 for 2L.

Some of the subjects in the present study had been extensively tested for DRs by Salorio-Corbetto et al. (2017). They performed a full TEN(HL) test and obtained Fast PTCs using several signal levels and frequencies. The estimates of *f*_e_ obtained by them (plotted relative to the reference values estimated here) are shown as asterisks in Figure 4. As noted earlier, in the present study, no estimates of *f*_tip_ could be obtained for ears 7L, 8L, and 8R. Salorio-Corbetto et al. (2017) reported that more runs than usual were needed for these ears to give stable results, but they were able to obtain Fast PTCs with clear minima. For ears 1R, 2R, 6L, 7R, and 8L, the estimates of *f*_tip_ obtained by Salorio-Corbetto et al. fell close to those obtained using the Smart DRT. However, for ears 1L, 7L, and 8R, there were marked deviations. For 1L and 7L, the estimates of Salorio-Corbetto et al. fell above those obtained here. This may have happened because they tested these ears 4 to 5 years ago, and the values of *f*_e_ may have shifted downwards over time.

Sixty catch trials (3 runs × 20 catch trials) were conducted for each ear. For 12 ears, the false positive rate was 3.3% or smaller, being 1.0% on average. Ears 5R and 1R gave false positive rates of 10% and 17%, respectively. Both subjects reported having tinnitus in their right ears.

**Figure 5.**
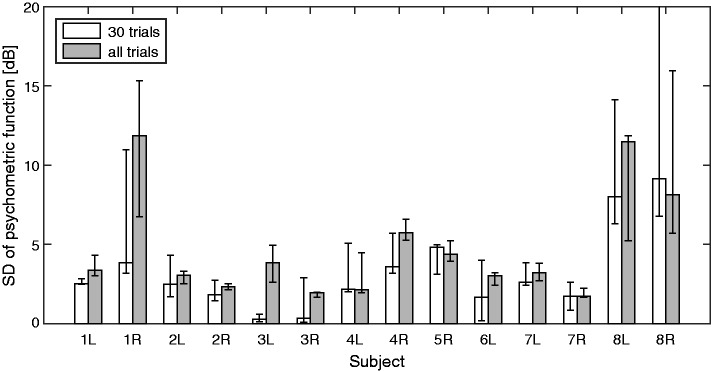
Estimated standard deviation of the psychometric function (a Gaussian CDF) for each ear after 30 trials (white bars) and 100 trials (gray bars). Bars show the median of the three runs, and error bars show the range.

It is useful to be able to check the consistency of the performance of a subject while the data are being collected. This can allow the test duration to be adjusted individually in order to achieve a predetermined level of accuracy. A possible method for doing this is to estimate the steepness of the psychometric function relating the proportion of “yes” responses to the sound level of the masker. This was done by expressing all masker levels relative to the PTC that was calculated from the most likely combination of *f*_e_ and OHCL( *f*_e_). Having removed the effect of frequency in this way, the hyperparameters of a Gaussian Process with a linear kernel in level and Gaussian CDF likelihood function were optimized (see Rasmussen & Williams, 2006). Positive responses for levels higher than 3 standard deviations above the normalized masker level and negative responses for levels lower than 3 standard deviations below the normalized masker level were considered as outliers and discarded before the optimization was repeated iteratively. Figure 5 shows the resulting estimates of the standard deviation of the psychometric function for each subject, with the bars depicting the median for the three runs and the error bars depicting the range. White bars show estimates after 30 trials, and gray bars show estimates after all 100 trials. In some cases, the estimates after 30 trials were much lower than after all 100 trials or even close to zero. This may happen when the estimated PTC perfectly separates the audible and inaudible trials, as was the case for ears 3L and 3R. For the majority of ears, the estimated standard deviation was between 2 and 4 dB and thus close to the 3 dB value that was used in the active-learning process. However, the standard deviations for ears 1R, 8L, and 8R were markedly higher.

**Figure 6.**
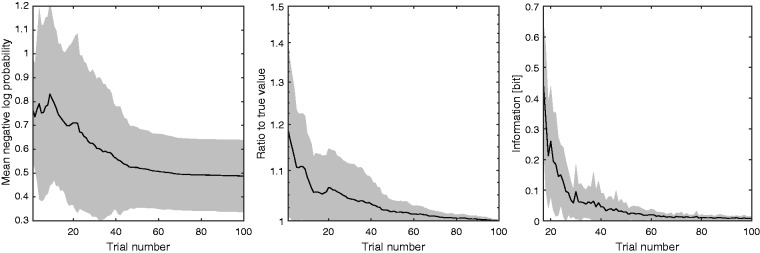
Mean negative (natural) log probability of predicting responses for all 100 trials correctly from the responses for the first *N* trials (left), ratio of f^*_e,n_*_ trials_/*f^_e_*_,100 trials_ or its reciprocal if smaller than 1 (middle), and the queried information (right) as a function of trial number. Solid lines show the mean across ears and runs, and gray areas show ±1 standard deviation. All three measures are close to asymptotic values after about 50 trials.

An important question for active-learning tests is the value of *N* needed to yield a reliable result. Figure 6 shows three measures of the accuracy of the fit as a function of *N*. The left panel shows the negative (natural) log probability of predicting the responses for all 100 trials correctly from the responses for the first *N* trials, divided by 100. The lower this number, the more accurate are the predictions. The middle panel shows the ratio between the most likely value of *f*_e_ after *N* trials and the most likely value after 100 trials, or its reciprocal if the ratio was smaller than 1. The right panel shows the mutual information that was queried in the *N*th trial, which declines from a theoretical maximum of 1 bit to 0.1 bit after about 25 trials, before reaching an asymptote of 0. For all panels, solid lines show the means across ears and runs, and gray areas show ±1 standard deviation. All measures are close to asymptotic values after about 50 trials. The mutual information (right panel) is available during a run, that is, it can be calculated with the knowledge that is available after the *N*th trial. It is highly correlated with the mean negative log probability, *r*(98) = 0.88, *p* < .001, and the ratio of the estimate of *f*_e_ to the true value, *r*(98) = 0.84, *p* < .001. Hence, the mutual information could be used to decide when *f*_e_ was determined with sufficient precision for a run to be terminated.

## Discussion

As shown in the bottom-right panel of Figure 1, a basal DR could start at a frequency where the audiometric threshold was only slightly higher than normal. More generally, the audiometric threshold at *f*_e_ varied widely across ears. Also, the slope of the audiogram for frequencies close to *f*_e_ varied widely across test ears. This is consistent with previous results showing that the presence and edge frequency of a DR cannot be diagnosed reliably from the audiogram (Aazh & Moore, 2007; Vinay & Moore, 2007).

The open symbols in Figure 2 show that the estimates of *f*_e_ from the three Smart DRT runs were close to each other, that is, the active-learning procedure led to reproducible results. This was the case even when the Fast PTCs failed to provide a clear result, although for the subjects for whom this was the case the Smart DRT results varied more across runs than for the other subjects. The estimates of *f*_e_ obtained using the Smart DRT were close to the estimates based on “successful” Fast PTCs, and within the range of plausible values of *f*_tip_ given the difficulty of estimating *f*_tip_ from the irregular PTCs. The similarity of the estimates of *f*_e_ for the Smart DRT and the Fast PTC methods suggests that the values estimated using the Smart DRT are close to the “true” values.

The estimates of *f*_e_ obtained by Salorio-Corbetto et al. (2017) on the basis of several Fast PTCs agreed with two of the three Smart DRT estimates in cases where the present Fast PTCs were not successful. Altogether, the results of the Smart DRT were consistent with estimates obtained using other procedures in 13 of 14 cases. For the remaining ear (8R), the Smart DRT runs indicated a need to be cautious in interpreting the results: Responses to the initial grid were markedly different across runs (not shown in the results section), suggesting that he was not consistent in his criterion for making a decision.

In the Smart DRT, the values of *f*_mask_ and *L*_mask_ for the next trial were chosen to provide information about *f*_e_ and OHCL( *f*_e_) but with some specific constraints to ensure exploration of a reasonable range of the parameter space. In theory, this is somewhat less efficient than choosing the parameters that provide the maximum information, but it prevented the model-based approach from becoming “stuck” in an inappropriate region of the parameter space and it ensured some randomness in the stimulus parameters for successive trials, preventing the model from overestimating the precision of its estimates of the model parameters.

The consistency of the performance of the subjects was determined using two measures, the false alarm rate and the steepness of the psychometric function. The two subjects who produced false positives at a rate of 10% or more both reported having tinnitus, and this may have contributed to their relatively high false-positive rates, if the pitch of their tinnitus was close to that of the signal (see also Lentz, Walker, Short, & Skinner, 2017). For the other subjects, the false alarm rate of 1% on average was exactly the fixed value that was applied in Equation 1. Thus, the active-learning procedure used reasonable assumptions for most subjects but was overconfident for the two outliers. In practice, it is difficult to adjust the false alarm rate to the value appropriate for an individual, since many trials are needed to achieve a reasonable estimate. Nevertheless interleaved catch trials can be used as a check to be evaluated at the end of a test.

For 11 of the 14 ears, the standard deviation of the Gaussian CDF used to estimate the psychometric function was reasonably close to the value of 3 dB that was assumed in Equation 1 (mean: 3.3 dB, range: 1.9–5.8 dB). However, the standard deviation of the psychometric function was considerably higher, at about 10 dB, for 1R, 8L, and 8R. The high value for 1R may have been a consequence of tinnitus. Nonetheless the final estimate of *f*_e_ for 1R was close to that obtained from the Fast PTCs and that obtained by Salorio-Corbetto et al. (2017). This suggests that the Smart DRT worked for him, but more trials were needed than for the other subjects to obtain the same accuracy. In order for the estimate of *f*_e_ to fall and stay within 0.3 Cams of the final estimate, 63, 50, and 45 trials were needed in his three runs, compared with the average of 33 trials. For 8L and 8R, the relatively flat psychometric functions may reflect his difficulty in distinguishing the tone from the noise. Some subjects with DRs hear a tone whose frequency falls within a DR as being noise-like (Huss & Moore, 2005). Cognitive factors are unlikely to account for the relatively flat psychometric functions, because his psychometric function for the audiogram was steeper than normal, showing a near-perfect separability of yes and no responses.

Ideally, the steepness of the psychometric function should be set to the individual value in Equation 1. However, its optimization is not an explicit aim of the Smart DRT and is not required to obtain a reasonable result. During early trials, perfect separability between yes and no responses may be achieved due to the data being sparsely spaced on the ( *f*_mask_, *L*_mask_) initial grid. After 30 trials, this was the case for both ears of Subject 3 (3L and 3R), and at least one run for 6L (see Figure 5). Underestimation of the standard deviation of the psychometric function leads to overconfidence in the current estimates of *f*_e_ and OHCL( *f*_e_). This can have two disadvantages. First, it may cause the procedure to stick to wrong estimates by preventing it from querying parameter values around the true values. Second, overconfidence leads to an underestimation of variance or uncertainty, which may be used as a criterion for stopping the test. A suitable strategy for optimizing the steepness of the psychometric function would be as follows. For the first few active-learning trials, set the standard deviation of the CDF to 3 dB. Afterwards, set the standard deviation to the estimated value with the restriction that it is at least 2 dB. If it is bigger than 20 dB, the run should be restarted.

The number of trials should be chosen to provide an estimate that is accurate enough for fitting a hearing aid. It has been recommended that amplification should not be provided for frequencies higher than about 1.7*f*_e_ (Baer, Moore, & Kluk, 2002). A precision of 0.3 Cams corresponds to a factor of less than 1.05 in frequency for frequencies between 500 and 4000 Hz. When this precision is reached, the cutoff frequency used for fitting would be between 1.6 and 1.8*f*_e_, which is accurate enough for an initial fitting. A ratio of about 1.05 or better was reached on average after about 30 trials (Figure 6, middle). The ratio was below 1.05 after 50 trials even for the worst cases. Using more than this number of trials did not increase the predictability of the entire data set (Figure 6, left). Fifty trials of the Smart DRT plus some catch trials and one run of the Quick TEN(HL) test were typically done in 5 to 8 min. The test could be even faster for some subjects if the stopping criterion was based on the information obtained in the next trial (Figure 6, right) or the uncertainty about *f*_e_ (see Figure 2). If this approach is adopted, the individual consistency of responses (Figure 5) should be taken into account, since it directly affects the uncertainty about *f*_e_.

## Conclusions

The present study proposed and evaluated an active-learning method, Smart DRT, for quickly detecting a basal DR and estimating its edge frequency, *f*_e_. The duration required for testing one ear was 5 to 8 min. The estimates of *f*_e_ were close to those obtained using other methods for 13 of 14 test ears, which is a slightly higher success rate than for Fast PTCs when the same amount of time was spent. The Smart DRT may be useful when fitting a hearing aid, providing accurate estimates of *f*_e_ in a short time.
